# Michigan State Board of Health

**Published:** 1881-09

**Authors:** 


### ARTICLE IX.

### MICHIGAN STATE BOARD OF HEALTH.

The regular meeting of this Board was held at Lansing, July 12, all the members being present, as follows: Hon. LeRoy Parker, of Flint; Rev. D. C. Jacokes, of Pontiac; Henry F. Lyster, M.D., of Detroit; J. H. Kellogg, M.D., of Battle Creek; Arthur Hazlewood, M.D, of Grand Rapids; John Avery, M.D., of Greenville; and Henry B. Baker, M.D., Secretary.

Hon. LeRoy Parker was elected President of the Board for the ensuing two years.

#### SMALL-POX.

Dr. Jacokes spoke of an immigrant tramp-burglar who came down with the small-pox while confined in the jail at Pontiac. He and another prisoner in the jail were removed to the temporary hospital. The prisoner stole the clothes of the immigrant, and, leaving his own, ran away. Some one then stole the prisoner's cast-off clothes and bedding after supposed disinfection, and by this means small-pox was communicated to more than sixteen persons. He also reported that a second immigrant brought small-pox near Pontiac, but the disease was restricted.

Dr. Kellogg reported that an immigrant, sick with small-pox, had recently been put off a M. C. R. R. train at Battle Creek. He remained about the depot all day before it was discovered that he had small-pox. He was then removed to a tent-hospital.

Dr. Henry reported an outbreak of small-pox, apparently brought by an immigrant Dane, who was vaccinated and not sick himself, to a camp in Montcalm county. The immigrant slept with and gave the disease to a countryman who was vaccinated before his arrival in this country, six years ago. The disease was light, the man not being confined to his bed at all, and finally he went to a family of five unvaccinated persons, all of whom had the disease lightly. There were other cases in the neighborhood coming from the same source. The immigrant probably brought the contagium in his clothing from some infected city or immigrant on the journey, as he said there was no small-pox on board the ship on which he came, though there were cases of diphtheria on board.

A communication was received from the American Public Health Association, asking the influence of this Board to secure legislation making it a criminal offense for any person to communicate any communicable disease, such as small pox, scarlet fever, or venereal diseases, and giving to boards of health and health officials the same power in the prevention and suppression of other diseases as they now possess in cases of small-pox.

The Secretary presented a resolution of the American Public Health Association, asking the Michigan Board to use its influence to secure general vaccination.

THE CHICAGO SANITARY CONFERENCE.

By direction of the Board, the Secretary had attended the conference of delegates from local and State boards of health, held at Chicago, June 29, for the purpose of devising means for preventing the introduction of small-pox and other diseases by immigrants. As Secretary of the Conference, he had prepared an official report and sent it to the National Board of Health for publication in its bulletin.

The action of the Sanitary Conference, to prevent the spread of small-pox, was indorsed, and resolutions were adopted requesting the National Board of Health to secure, if possible, the vaccination of immigrants before they land in this country; asking the attention of every local board of health in Michigan to the details of the plan adopted at the late Chicago conference; calling upon them to secure a careful inspection of all immigrants entering and remaining within their jurisdiction, and a prompt vaccination or re-vaccination with pure and fresh bovine virus of all persons not protected against small-pox; calling attention to the need of establishing a quarantine at Port Huron; also, asking the National Board of Health to aid in preventing the introduction of small-pox and other communicable diseases by immigrants landing at eastern ports.

Dr. Baker read resolutions of the Tennessee State Board of Health, indorsing the action of the Memphis Board of Health, commending the inspection service of the National Board of Health as being very much more effective than local quarantines, with less detention and annoyance to commerce.

### VACCINATION FOR VARIOUS DISEASES.

Dr. Lyster, Committee on Epidemics and other Diseases, read a translation of two important papers recently published in France, on the causation of certain communicable diseases, which gave details of successful methods of making viruses which can be used in vaccination, and are effective in preventing deaths from these diseases. He received the thanks of the board, and was requested to embody his remarks and so much of the translation as was essential, in a paper for publication.

Dr. Baker had paid some attention to the same subject in connection with diseases of animals, affecting the public health. He mentioned a paper by Prof. Law, of Cornell University, suggesting that these protective viruses all seemed to be made in accordance with a general law, namely, by their cultivation in fluids with access of free oxygen, and this gives us great hope of soon being able to make protective vaccination for many of the most dangerous diseases in animals and mankind.

Dr. Baker reported the investigation of an outbreak of a new disease in England, traced to the eating of American hams. The cause of the disease proved to be a virus which was used to inoculate animals of various kinds and reproduced the same disease in them. From the accounts it seems probable that it is no more nor less than our hog cholera. The symptoms closely resemble in some respects the disease known last winter in this country as "winter cholera."

### SANITARY ASSOCIATIONS.

Dr. Jacokes referred to the Pontiac Sanitary Association, and the work it was doing for public health in that city.

Dr. Kellogg reported the formation of a sanitary association at Battle Creek, as a fruit of the recent sanitary convention held there by this board. Among the subjects brought before the association was that of impure water. He had examined a sample of water used at an eating-house, among the boarders of which there were seven cases of typhoid fever last year. It contained a large amount of organic matter. Also a sample containing organic matter and a large amount of chloride of sodium, used by a family in which there had been much illness.

The request of the sanitary convention at Battle Creek, that this board issue a circular on criminal abortion, was referred to Dr. Kellogg as special committee.

### FUTURE PUBLICATIONS.

Drs. Lyster and Baker reported their revision of the document on the Restriction and Prevention of Diphtheria, and different points were discussed, amended and the document adopted. Thirty thousand copies will be printed and the document stereotyped, so that local boards of health may secure any number of copies at cost of paper and presswork.

Dr. Jacokes referred to the great lack of knowledge among those who ought to know, as to what constitutes thorough disinfection. He proposed to remedy this by the preparation of a circular on disinfection.

Drs. Baker and Kellogg were appointed a special committee to prepare a tract on disinfection, which shall give the best method adapted to each disease and to each article to be disinfected, and which shall call attention to the many useless substances now employed for such purposes.

The document heretofore issued on the treatment of the drowned being out of print, it was referred to a committee of revision, with a view to its republication.

Dr. Baker was instructed to prepare a paper on the best-methods of constructing hospitals for communicable diseases, avoiding the use of the name "pest-house."

### LEGISLATION UPON HEALTH.

Hon. Leroy Parker, Committee on Legislation, made a report relative to public health acts passed by the last legislature, giving the titles of forty-eight acts bearing directly or indirectly on public health subjects, mentioning the subject of each act. These acts give increased powers to local boards of health, additional appropriations to the State Board of Health, authorize the board to control State swamp lands to appropriate lands to drain overflowed lands, etc.

The recommendation by Dr. Foster Pratt, of Kalamazoo, for the amendment of the law relative to reporting dangerous diseases, was referred to the Committee on Legislation.

Under the new appropriation made by the recent legislature, the board authorized the purchase of additional meteorological instruments for the use of the board's observers in different parts of the State

WORK IN THE OFFICE.

The Secretary read a report of work in the office, which included a statement of a number of health officers appointed for the present year, beginning April 1, as follows: In townships, 797; villages, 107; cities, 32; total, 936. For the year ending April 1, there were appointed, for townships, 827; villages, 112; cities, 36; total, 975—a difference of 39. It is believed that more than 39 will be returned before the close of this year. The 975 health officers appointed for 1880 made annual reports to this board, as follows: Of townships, 414; of villages, 55: of cities, 14. Clerks of local boards of health have sent annual reports for 1880: From townships, 501; from villages, 36; from cities 7. The compilation of these annual reports is now in progress.

During the quarter the correspondence of the office has been materially increased. This was partly caused by the outbreaks of small-pox in the State, and by the prevalence in some places of diphtheria. In one northern township, where the health officer is not a physician, the local officers applied to this office for a physician to be sent to aid in stopping the spread of the disease; and by direction of the Secretary of the State Board, Dr. Hawkhurst, ex-health officer of West Bay City, went there at the expense of the township, and has reported that the outbreak has been stopped.

The usual number of complaints have been received of sickness caused by flooding rivers for the purpose of running logs in the northern part of the State. In answering these the Secretary has used Mr. Parker's paper on the powers and duties of local boards of health.

### EXAMINATION IN SANITARY SCIENCE.

The fee for examination in sanitary science was changed from $10 to $1, the latter sum being deemed sufficient to cover the actual expense. It was voted that applicants unable to be present at this meeting may be examined at the meeting of the Board, October 11, 1881, application to be made to the Secretary, at Lansing.

### GLUCOSE SUGAR AND SYRUP.

Several samples of sugar and syrup, manufactured at the Michigan Grape and Sugar Factory, at Detroit, were presented to the Board and partially examined.

### NOTICES OF CONTAGIOUS DISEASES TO SCHOOLS.

The Secretary presented samples of notices of contagious and infectious diseases sent by the health officer of Grand Rapids and Tecumseh to the superintendents of schools in those cities, and suggested that if the officer of each city would send such notices to superintendents, it would be a very important public health measure.

### PREVENTION OF BOILER EXPLOSIONS.

An account of an experimental boiler explosion, by Dr. T. Lawson, was presented. His view is, that they can be prevented by such a construction of the boiler as will stop the too rapid increase of steam under suddenly-reduced pressure, as at starting the engine, or by the sudden introduction of cold water. Results thus far seem to demonstrate the correctness of his theory.

The Secretary was directed to present hereafter, at each meeting of the Board, a *résumé* of the action of other State boards of health.

After auditing bills and accounts, etc., the Board adjourned, to meet October 11, 1881.

